# UPEC Colonic-Virulence and Urovirulence Are Blunted by Proanthocyanidins-Rich Cranberry Extract Microbial Metabolites in a Gut Model and a 3D Tissue-Engineered Urothelium

**DOI:** 10.1128/spectrum.02432-21

**Published:** 2022-08-16

**Authors:** Charlène Roussel, Stéphane Chabaud, Jacob Lessard-Lord, Valentina Cattero, Félix-Antoine Pellerin, Perrine Feutry, Valérie Bochard, Stéphane Bolduc, Yves Desjardins

**Affiliations:** a Institute of Nutrition and Functional Foods (INAF), Faculty of Agriculture and Food Sciences, Laval University, Québec, Quebec, Canada; b Centre de Recherche en Organogenèse Expérimentale de l Université Laval/LOEX, Centre de Recherche du CHU de Québec‐Université Laval, Axe Médecine Régénératrice, Québec, Quebec, Canada; c Diana Food, Rennes, France; University of Guelph

**Keywords:** PAC, cranberry, gut metabolome, UPEC, urinary tract infections, urothelium, virulence genes

## Abstract

Uropathogenic Escherichia coli (UPEC) ecology-pathophysiology from the gut reservoir to its urothelium infection site is poorly understood, resulting in equivocal benefits in the use of cranberry as prophylaxis against urinary tract infections. To add further understanding from the previous findings on PAC antiadhesive properties against UPEC, we assessed in this study the effects of proanthocyanidins (PAC) rich cranberry extract microbial metabolites on UTI89 virulence and fitness in contrasting ecological UPEC’s environments. For this purpose, we developed an original model combining a colonic fermentation system (SHIME) with a dialysis cassette device enclosing UPEC and a 3D tissue-engineered urothelium. Two healthy fecal donors inoculated the colons. Dialysis cassettes containing 7log_10_ CFU/mL UTI89 were immersed for 2h in the SHIME colons to assess the effect of untreated (7-day control diet)/treated (14-day PAC-rich extract) metabolomes on UPEC behavior. Engineered urothelium were then infected with dialysates containing UPEC for 6 h. This work demonstrated for the first time that in the control fecal microbiota condition without added PAC, the UPEC virulence genes were activated upstream the infection site, in the gut. However, PAC microbial-derived cranberry metabolites displayed a remarkable propensity to blunt activation of genes encoding toxin, adhesin/invasins in the gut and on the urothelium, in a donor-dependent manner. Variability in subjects’ gut microbiota and ensuing contrasting cranberry PAC metabolism affects UPEC virulence and should be taken into consideration when designing cranberry efficacy clinical trials.

**IMPORTANCE** Uropathogenic Escherichia coli (UPEC) are the primary cause of recurrent urinary tract infections (UTI). The poor understanding of UPEC ecology-pathophysiology from its reservoir–the gut, to its infection site–the urothelium, partly explains the inadequate and abusive use of antibiotics to treat UTI, which leads to a dramatic upsurge in antibiotic-resistance cases. In this context, we evaluated the effect of a cranberry proanthocyanidins (PAC)-rich extract on the UPEC survival and virulence in a bipartite model of a gut microbial environment and a 3D urothelium model. We demonstrated that PAC-rich cranberry extract microbial metabolites significantly blunt activation of UPEC virulence genes at an early stage in the gut reservoir. We also showed that altered virulence in the gut affects infectivity on the urothelium in a microbiota-dependent manner. Among the possible mechanisms, we surmise that specific microbial PAC metabolites may attenuate UPEC virulence, thereby explaining the preventative, yet contentious properties of cranberry against UTI.

## INTRODUCTION

Urinary tract infections (UTI) in human are generally caused by uropathogenic Escherichia coli (UPEC). More than 50% of women will experience symptomatic UTI during their lifetime. Among them, 1/3 will develop recurrent UTIs (1). UPEC life cycle starts in the intestinal niche, where the bacterium naturally cohabits with the gut microbiota (2, 3). The fecal-periurethral contamination promotes UPEC tropism and colonization through the urinary tract and bladder (3, 4). At this site, UPEC utilizes numerous virulence (e.g., adhesins, toxins) and fitness factors (e.g., iron acquisition system) to adhere, disseminate within the mucosa and ultimately cause disease (4). There is an evident lack of understanding of UPEC ecology-pathophysiology in the gut reservoir, with studies focusing mainly on the infection site. Elucidating the distinct UPEC lifestyles (e.g., survival, virulence genes expression) from the gut to the urinary tract is crucial to add knowledges from previous works that were dedicated to study UTI pathogenesis, recurrence mechanisms, and to improve treatment effectiveness.

So far, antibiotics remain the standard prophylaxis and treatment, contributing to the drastic rise of multidrug-resistant uropathogens and their concomitant health repercussions (5). Thus, there is a pressing need for the development of more effective prophylaxis to address the UTI societal burden and limit the use of antibiotics. Positioning cranberries consumption (Vaccinium macrocarpon Aiton) as a natural and nonantibiotic approach meets consumers’ demand for integral alternatives to prevent the development of the infections. Although cranberry is extensively recommended for UTIs prophylaxis (6), there is discrepancy and controversial results between studies with underlying mechanisms of action that are still open to debate. Cranberry is composed predominantly of water, followed by a complex mixture of organic acids, fructose and polyphenols including flavonoids, anthocyanidins, proanthocyanidins (PAC), catechins, and triterpenoids (7). The whole cranberry fruit or specific fractions have been studied for their potential role in inhibiting UPEC adhesion/invasion to the urinary tract, limiting biofilm formation, and/or in reducing clinical symptoms or UTI events (6, 8–15). In particular, many research evidence shows that cranberry PAC are the most clinically relevant component in preventing UTI in women (8). PAC display a limited absorption in the intestine and are extensively metabolized in the colon by the gut microbiota (12, 16). It is now believed that PAC antiadhesive properties on UPEC in bladder are more likely caused by their absorbed microbial metabolites, as well as for the reduction of biofilm formation or clinical symptoms (13, 14). Among the PAC microbial metabolites, conjugates (O-methyl ethers and sulfates) of phenolic acids (phenylpropionic, phenylvaleric, phenylacetic, benzoic, and cinnamic acids) (13), and other microbial metabolites, including phenyl-γ-valerolactones (17), have been associated with the preventive actions of cranberry flavonoids and phenolic acids on UTI.

To contribute to further understanding from the previous findings on PAC antiadhesive properties against UPEC, the present work aims to assess the effect of gut microbial metabolites of PAC-rich cranberry extract on UPEC virulence and fitness pathways. For this purpose, we developed an original model combining an artificial colonic fermentation system (SHIME) with a dialysis cassette device and a 3D tissue-engineered urothelium coculture model.

## RESULTS

### Metabolomes of donors A and B display a differential PAC catabolism in the colon.

**FIG 1:**
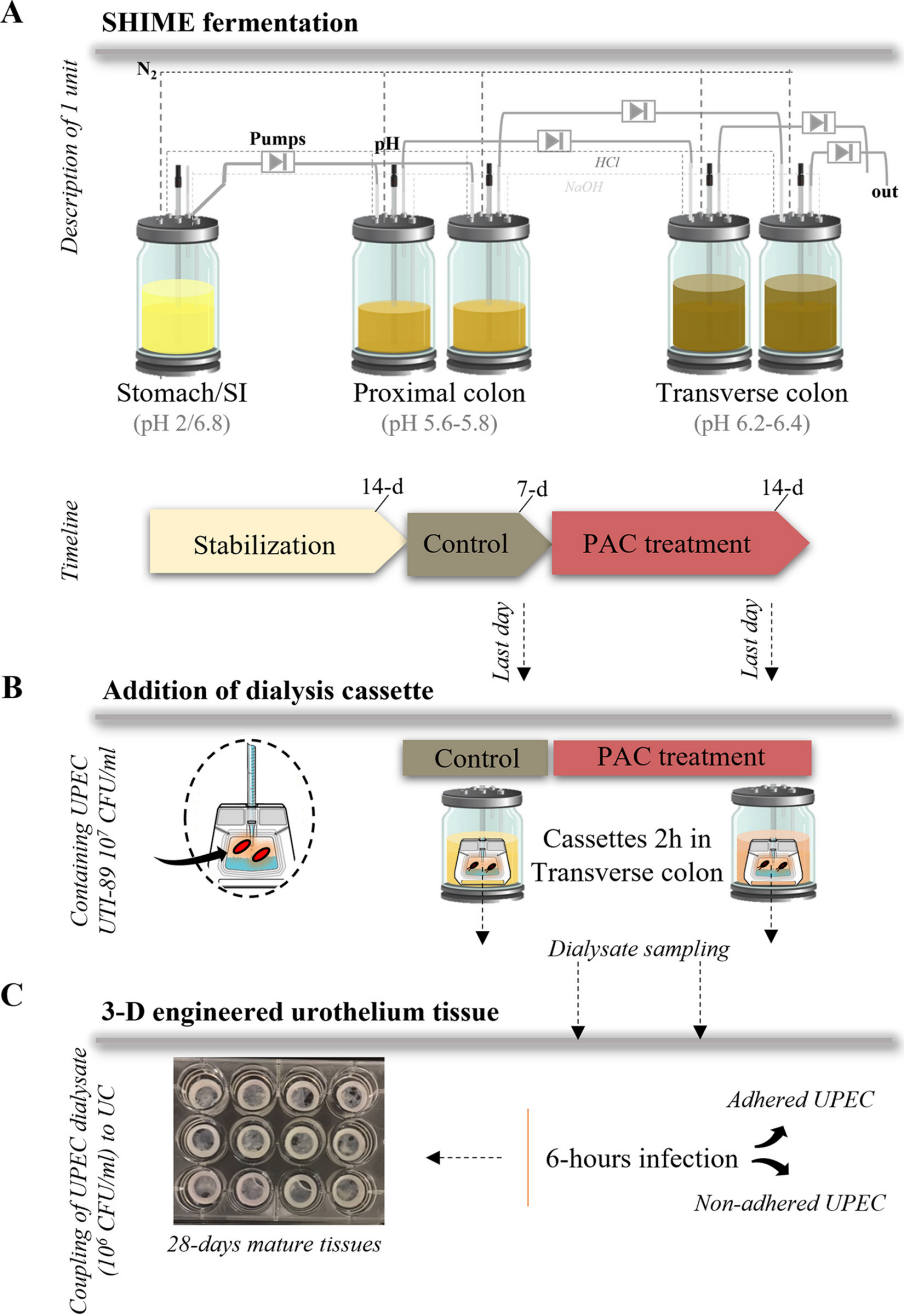
Study design. (A) Illustration of one unit of the TWIN-SHIME fermentation system, including reactors in series from the stomach/small intestine (SI) to the transverse colon. The colonic reactors of each unit were inoculated with two different fecal donors, both in duplicates. The three phases and duration of fermentation are shown in the timeline. The treatment consisted of the addition of 86.8 mg PAC-rich cranberry extract/day in the SHIME stomach for 14 days, consecutively catabolized in the proximal and transverse colon. (B) The transverse colon was chosen as site of interest for UPEC reservoir and final PAC catabolism. Therefore, dialysis cassettes (10 kDa) containing 7log_10_ CFU/mL UTI89 were added for 2h in the transverse colon to assess the effect of untreated (control)/treated (PAC) metabolome on UPEC behavior. After exposure, dialysates containing UPEC were stored until processing. (C) Dialysates containing UPEC (adjusted concentration of 6log_10_ CFU/mL UTI89) were then combined to a 3D tissue-engineered urothelium (UC) to recreate a 6-h infection period.

**FIG 2:**
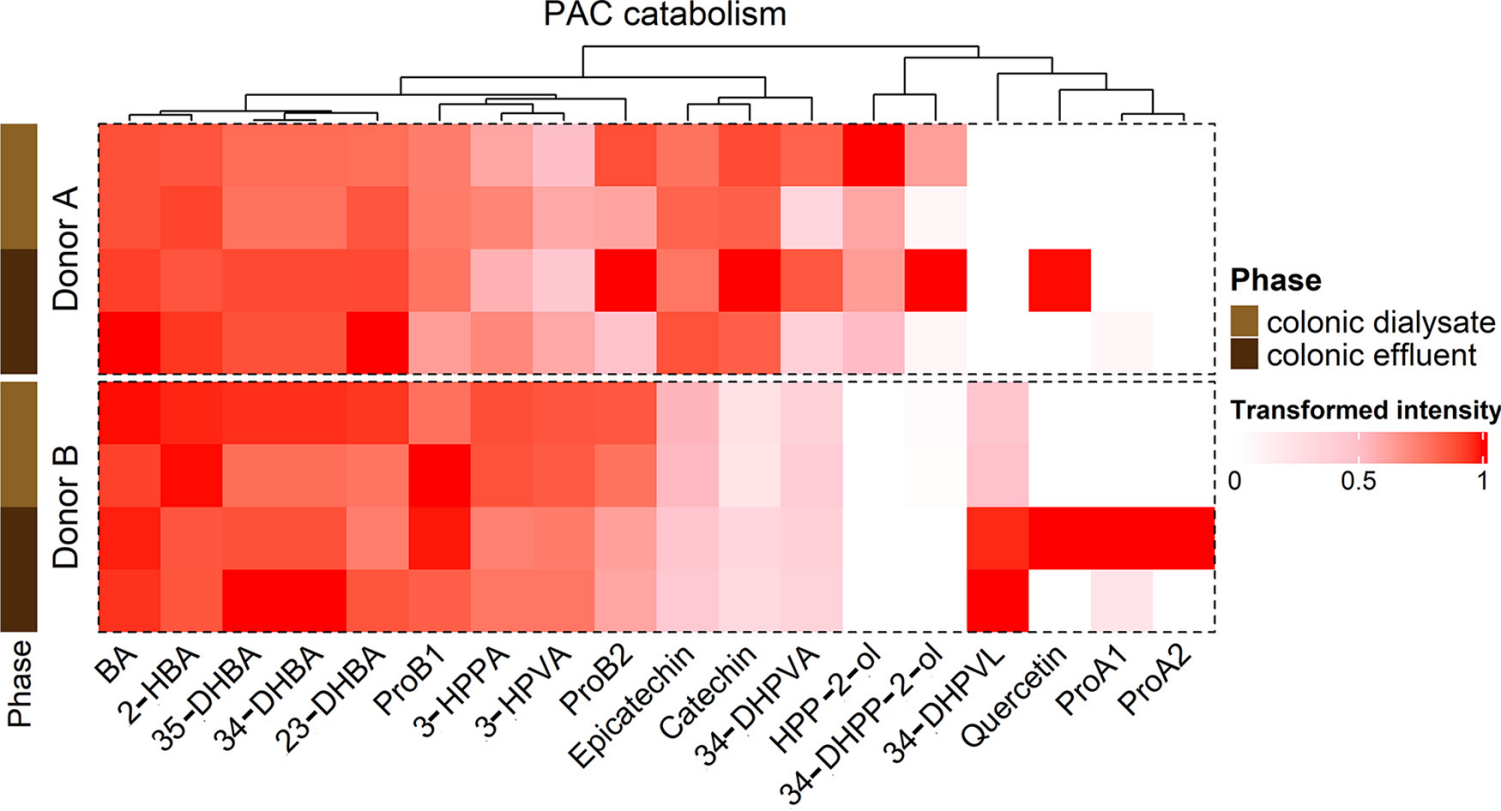
Hierarchical profiling of PAC catabolism between donors in gut effluents and dialysates after a 14-day treatment. Heatmap displaying the transformed intensity of the PAC catabolites remaining in gut effluents and dialysates after 14 days of PAC-rich extract treatment and following a 2-h UPEC exposure, as determined by UPLC-QToF in negative ionization. BA = Benzoic Acid; 3-HPVA = 5-(3′-hydroxyphenyl)valeric acid; 35-DHBA = 3,5-dihydroxybenzoic acid; ProA2 = Procyanidin A2; ProB2 = Procyanidin B2; HPP-2-ol = 1-(Hydroxyphenyl)-(2′,4′,6′-trihydroxyphenyl)-propan-2-ol; 2-HBA = 2-hydroxybenzoic acid; 3-HPPA = 3-(3′-hydroxyphenyl)propanoic acid;, 4-DHPVA = 5-(3′,4′-dihydroxyphenyl)valeric acid; ProA1 = Procyanidin A1; 34-DHPP-2-ol = 1-(Dihydroxyphenyl)-3-(2′,4′,6′-trihydroxyphenyl)-propan-2-ol; ProB1 = Procyanidin B1; 34-DHPVL = 5-(3′,4′-dihydroxyphenyl)-γ-valerolactone; 34-DHBA = 3,4-dihydroxybenzoic acid; 4_HPAA = 2-(4′-hydroxyphenyl)acetic acid; 34-DHPPA = 3-(3′,4′-dihydroxyphenyl)propanoic acid; 4-HPPA = 3-(4′-hydroxyphenyl)propanoic acid.

UPEC enclosed in dialysis cassettes were first exposed for 2-h to the metabolic environment of the SHIME transverse colon from two different donors after a control period and a 14-day PAC treatment (Fig. 1A and B). As proof of the efficacy of the dialysis device, most PAC metabolites present in colonic effluents were also found in colonic dialysate (Fig. 2). No PAC metabolites were found in the absence of microbiota. Differential production of PAC metabolites was seen between donors A and B. Donor B produced 34-DHPVL, ProA1 and ProA2, while donor A did not (Fig. 2). On the contrary, donor A produced HPP-2-ol and DHPP-2-ol. Finally, donor A predominantly produced catechin, epicatechin and 3,4-DHPVA compared to donor B.

### PAC treatment significantly affects UPEC growth in a donor-dependent manner.

**FIG 3:**
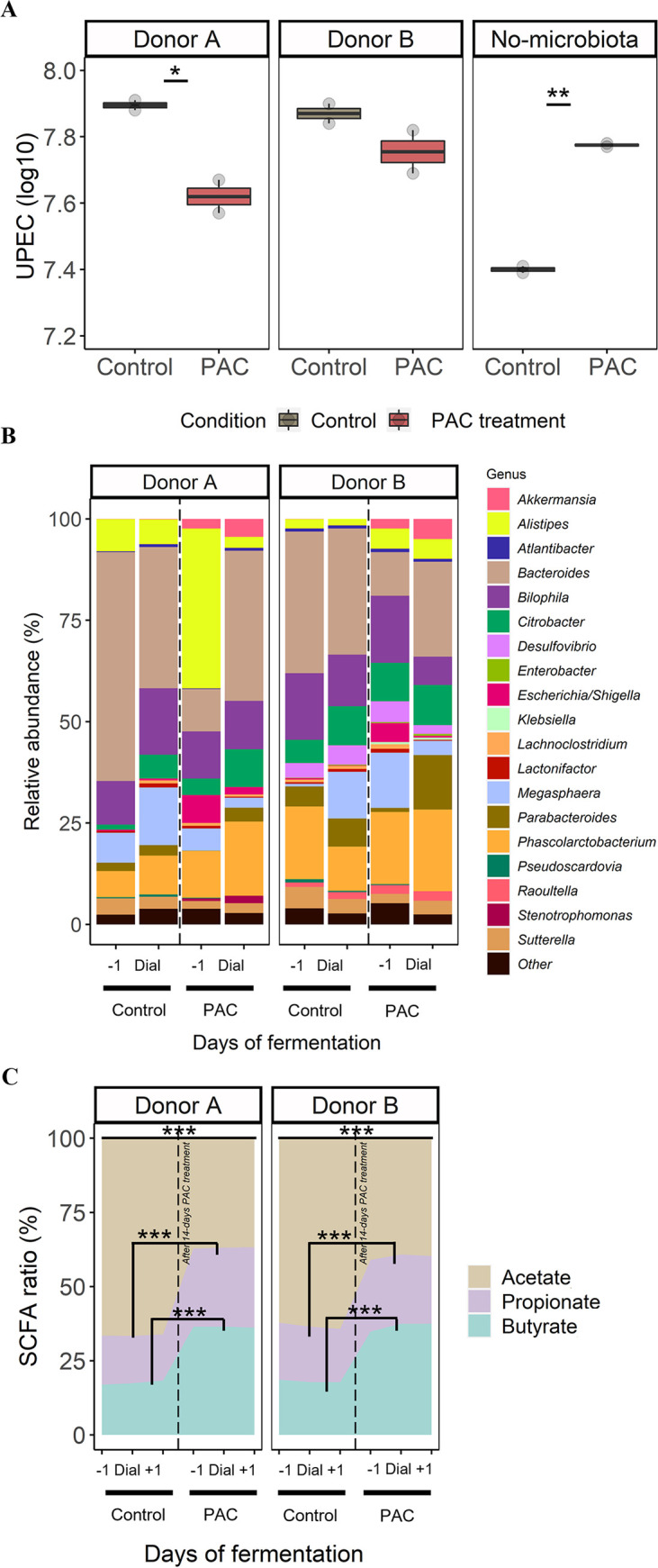
TWIN-SHIME combining dialysis cassette device as a model of short-term UPEC’s gut reservoir (A) Number of viable-culturable UPEC (log_10_ CFU/mL) remaining in the dialysate after a 2-h exposure with the PAC treated/non-treated metabolome of the transverse colon. A condition “no microbiota” was also performed by introducing dialysis cassettes in jars with digestive medium deprived of microbiota. Significant differences between PAC treatment and control are indicated with *P ≤ *0.05 (*) or *P ≤ *0.01 (**), as determined by the Friedman *post hoc* Wilcoxon test. (B) The microbial composition (%) of the 20 most abundant genera is shown. “Dial” represents the day of addition of dialysis cassettes containing UPEC in transverse colons of donors A and B, both during the control and PAC treatment. (C) Ratio-profile of short chain fatty acids (SCFA) for each treatment condition: before (-1 day), during addition of dialysis cassette “Dial,” and after addition (+1 day). Significant differences in production of acetate, butyrate and propionate were found between PAC treatment and control, *P ≤ *0.001 (***), as determined by the Friedman *post hoc* Wilcoxon test.

The gut metabolome of donor A caused a significant decrease in UPEC titer under PAC treatment (*P ≤ *0.05) compared to donor B (Fig. 3A, Fig. S2 in the supplemental material). Such differential response might be explained by variability in PAC catabolism as shown in Fig. 2, and therefore, depends on microbiota composition (Fig. 3B). Although PAC treatment stimulated the growth of *Akkermansia* in both donors, donor A was characterized by an upsurge of *Stenotrophomonas*, *Escherichia-Shigella,* and a decrease of *Megasphaera*, while donor B displayed a decrease of *Bacteroides* under PAC treatment (Fig. 3B). The fermentation by-products were also measured under both conditions. PAC treatment induced (*P ≤ *0.001) a sharp increase of butyrate and propionate in the transverse colon of both donors (Fig. 3C). Finally, in the absence of a microbiota, PAC treatment significantly stimulated UPEC growth (Fig. 3A). This suggests that the microbiota and microbial PAC metabolites blunt UPEC growth.

**FIG 4:**
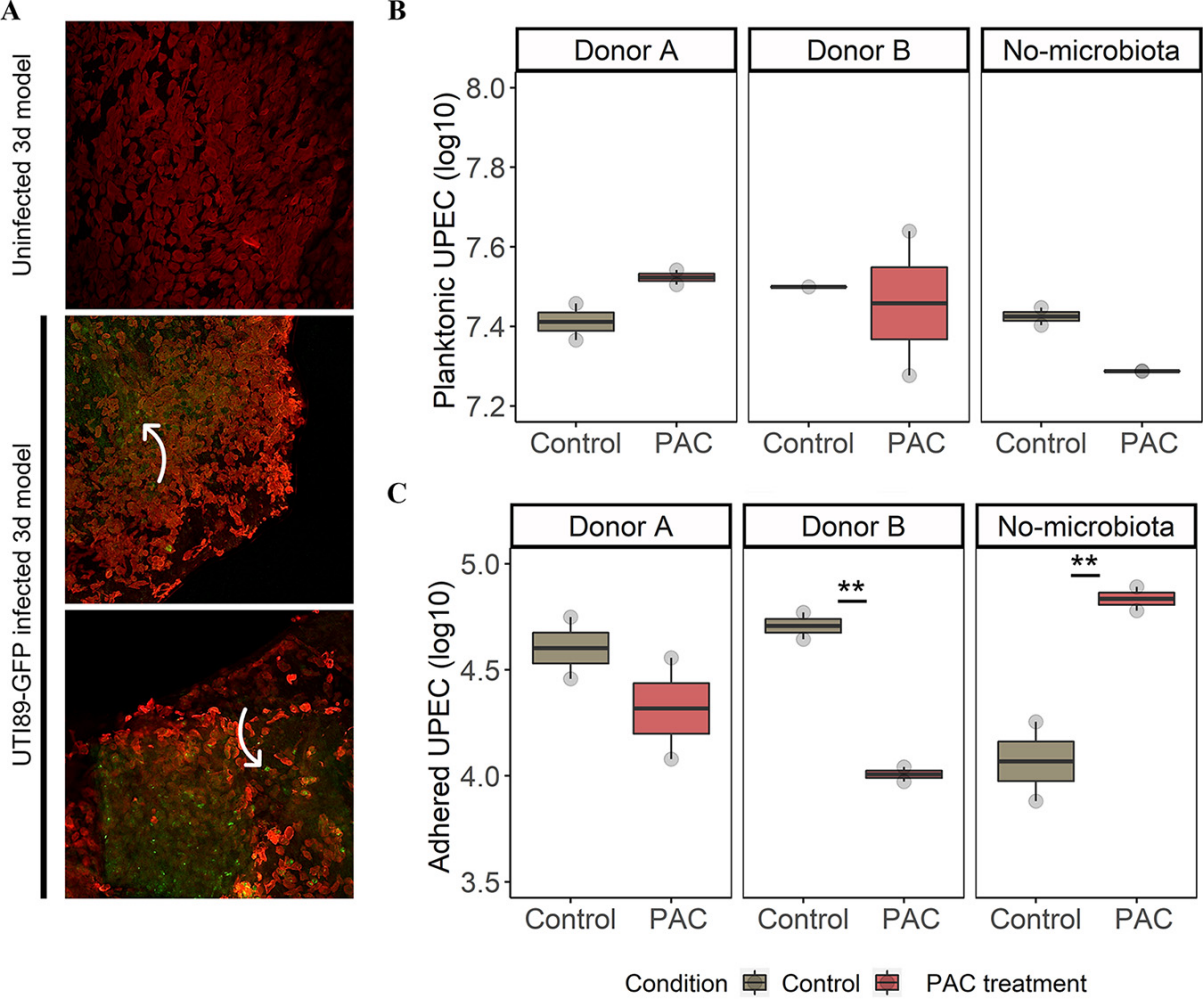
Engineered bladder mucosa as a model of acute urinary tract infection. (A) Bladder mucosa were reconstructed and infected by UPEC dialysates. Tissues were fixed and immunolabelled 6 h after infection to detect cytokeratins (red fluoresecnce, urothelial cells). UPEC were detected by GFP (green) fluorescence. Large number of bacteria at the surface or in the urothelial cells were visible and indicated by white arrows. (B) Number of viable-culturable UPEC (log_10_ CFU/mL) unattached to UC (planktonic) after a 6-h infection. (C) Number of viable-culturable UPEC (log_10_ CFU/mL) remaining attached to UC after a 6-h infection. A condition “no microbiota” was also performed by testing UPEC dialysate not exposed to the gut microbiota. Significant differences between PAC treatment and control are indicated with *P ≤ *0.01 (**), as determined by the Friedman *post hoc* Wilcoxon test.

Next, UPEC dialysates were used to infect urothelial cells (UC) (Fig. 1C, Fig. 4A). After a 6-h infection, cells were washed, and two UPEC ecosystems were analyzed: planktonic and adhered UPEC (Fig. 4B and C). No change in the number of planktonic UPEC was observed between control versus PAC (Fig. 4B). Under all conditions, a low proportion of UPEC adhered persistently onto UC, approximately 4–5 log_10_ (Fig. 4C). In this fraction, UPEC previously exposed to PAC metabolites from donor B displayed a significantly lower adhesion onto UC (4 log_10_) compared to the control (4.5 log_10_) (*P ≤ *0.01) (Fig. 4C). On the contrary, the lack of microbiota under PAC treatment stimulated the UPEC adhesion, while lowering the planktonic fraction.

### UPEC virulence genes are activated at the urothelium site and upstream in the gut reservoir.

**FIG 5:**
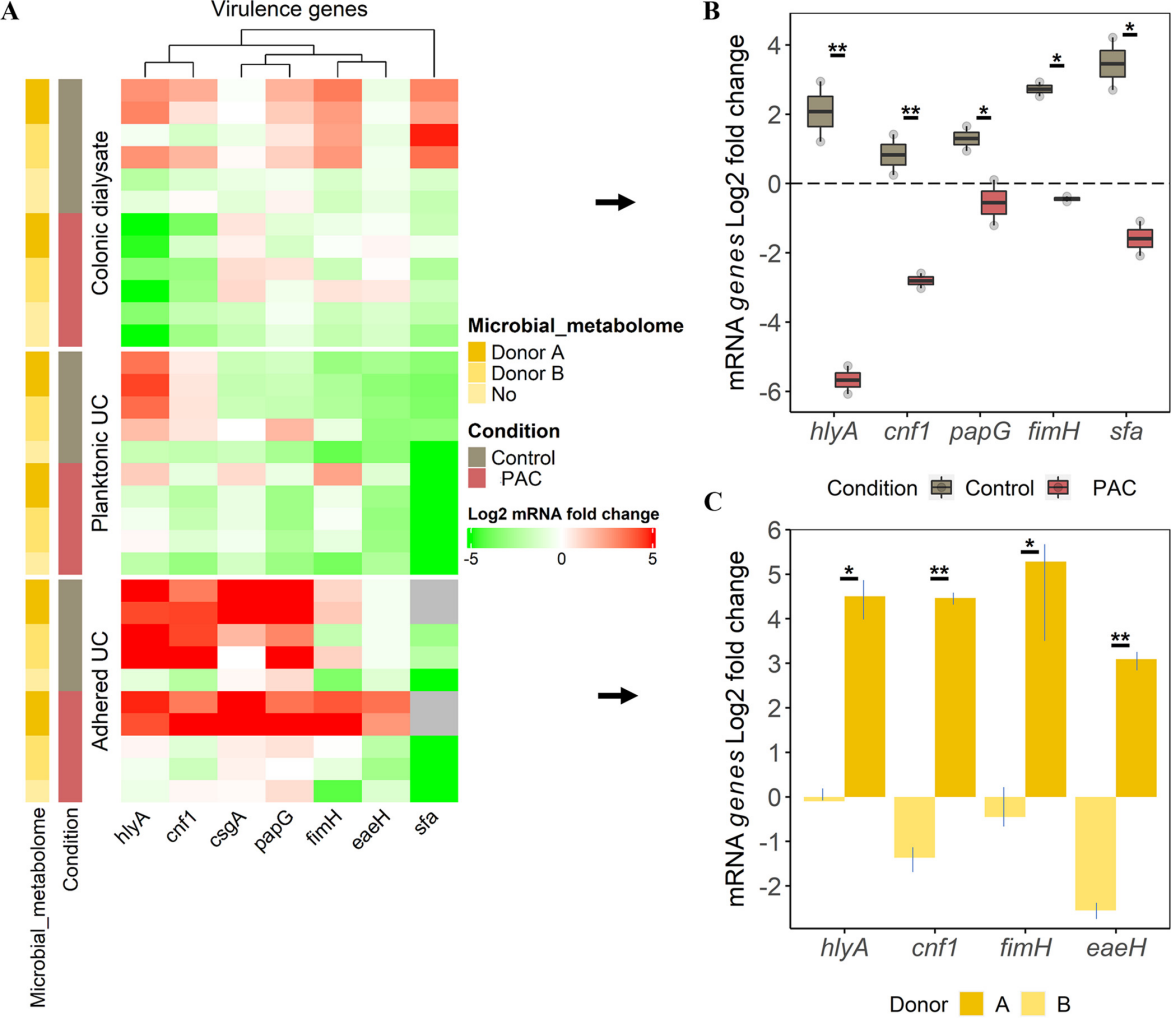
Profiling of UPEC main virulence genes expression from the gut reservoir to urothelium. (A) Heatmap displaying the log_2_ fold change UPEC virulence genes expression according to the ecosystem exposure (colonic dialysate, planktonic and adhered bacteria to UC), the microbial metabolome origin and the treatment. Induction (log_2_ fold change expression ≥ 1) is denoted in shade of red, and repression (≤ -1) in shade of green, as determined by RT-qPCR. Samples with failed amplification are displayed in gray. (B) Selection of genes from dialysis cassettes remaining significantly different between control and PAC conditions are indicated with *P ≤ *0.05 (*) or *P ≤ *0.01 (**), as determined by the Friedman *post hoc* Wilcoxon test. (C) Selection of genes from adhered UPEC treated with PAC that are significantly different between donors A and B, as determined by the Friedman *post hoc* Wilcoxon test.

Key virulence genes specific to UPEC strain UTI89 were monitored at the end of the UPEC incubation period in each ecosystem studied (after 2 h in gut dialysate and after 6 h onto UC) under both control (PAC free) and PAC treatment. Results were expressed in log_2_ expression fold changes (FC). UPEC virulence genes expression signatures were specific to the ecosystem studied (e.g., gut dialysate, planktonic UC, adhered UC) (Fig. 5A). In the control condition, most of the virulence genes encoding for toxins production and adhesins were expressed at the expected site of action: when adhered onto UC. Strikingly, these genes were already activated at an earlier stage in the colonic dialysate (Fig. 5A). However, in the absence of a microbiota, no virulence genes were activated.

### PAC treatment significantly impeded UPEC virulence genes activation in the gut reservoir.

Under PAC treatment, most of the virulence genes were downregulated in UPEC dialysate and planktonic UC compared to the control, regardless of the microbial metabolome origin (Fig. 5A). However, significant differences between control and treatment were noted for UPEC dialysate only (Fig. 5B). The genes encoding for toxins production including alpha hemolysin (*hlyA*) and cytotoxic necrotizing factor 1 (*cnf1*) were highly repressed (*P ≤ *0.01) under PAC with -5.8 log_2_ FC and -3 log_2_ FC versus 2 log_2_ FC and 1 log_2_ FC under control, respectively (Fig. 5B). Still in UPEC dialysate, genes encoding for adhesins/invasins, such as pili associated pyelonephritis (*papG*), type 1 fimbrial adhesin (*fimH*) and S fimbriae (*Sfa*), were significantly downregulated (*P ≤ *0.05). For instance, a 2.5 log_2_ FC expression was observed for *fimH* under control versus -0.5 log_2_ FC under PAC condition (Fig. 5B).

Adhered UPEC onto UC displayed a particular expression pattern under PAC treatment that appeared dependent on the microbial metabolome origin (Fig. 5A). Indeed, UPEC previously exposed to the PAC-treated metabolome of donor A (Fig. 2) showed significant overexpression of *hlyA*, *cnf1*, *fimH* and the gene encoding for attaching and effacing protein (*eaeH*) when adhering to urothelium, compared to donor B (Fig. 5C).

### PAC treatment significantly activates UPEC iron-acquisition genes in the gut reservoir.

**FIG 6:**
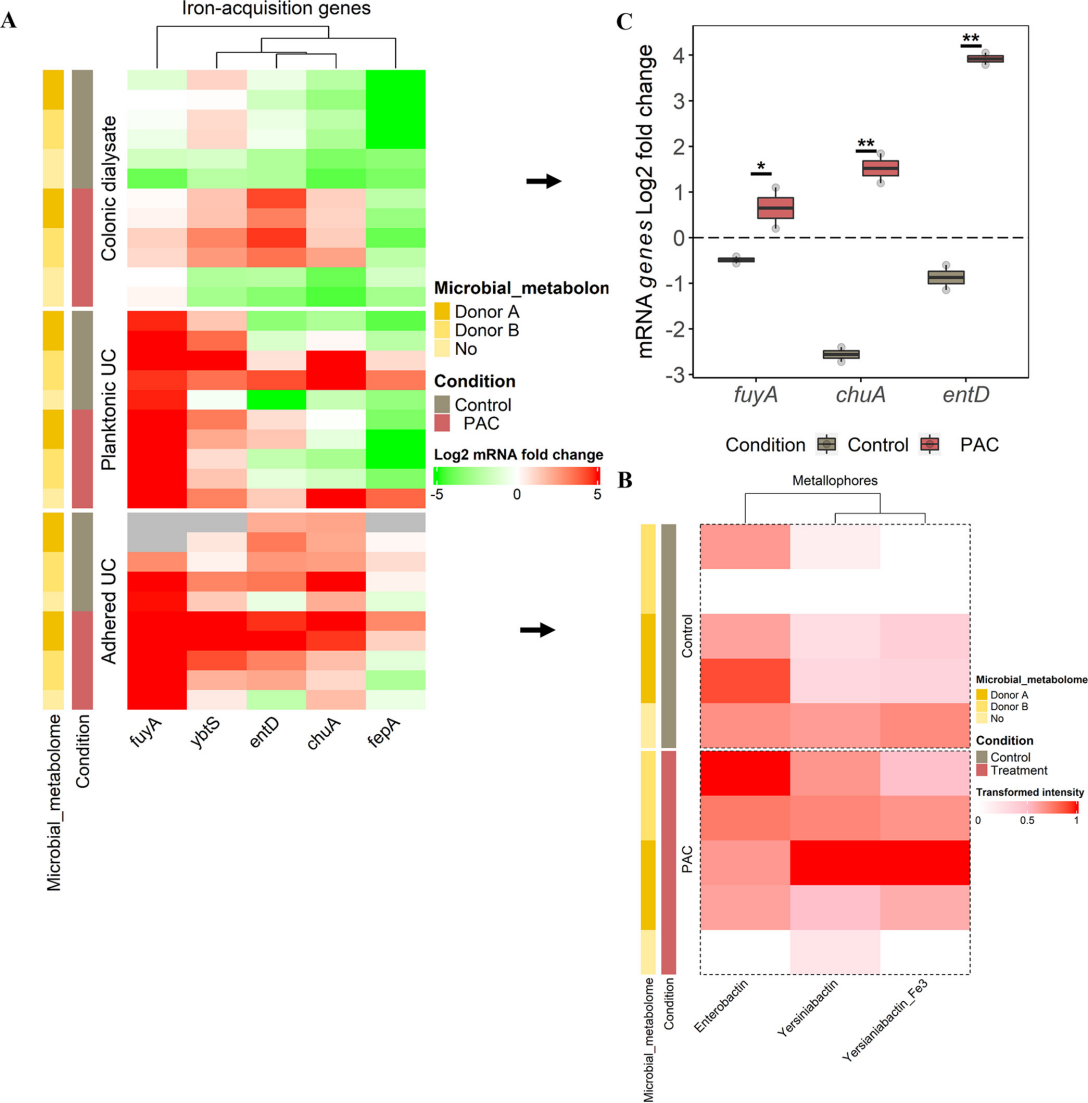
Profiling of UPEC iron-acquisition genes expression from the gut reservoir to urothelium. (A) Heatmap displaying the log_2_ fold change UPEC iron-acquisition genes expression according to the ecosystem exposure (colonic dialysate, planktonic and adhered bacteria to UC), the microbial metabolome origin and the treatment. Induction (log_2_ fold change expression ≥ 1) is denoted in shade of red and repression (≤ -1) in shade of green, as determined by RT-qPCR. Samples with failed amplification are displayed in gray. (B) Heatmap displaying the transformed intensity of the metallophores metabolites remaining in UC medium after 6 h of UPEC infection, as determined by Reverse-Phase Liquid Chromatography (RPLC) positive. (C) Selection of genes from dialysis cassettes remaining significantly different between control and PAC conditions are indicated with *P ≤ *0.05 (*) or *P ≤ *0.01 (**), as determined by the Friedman *post hoc* Wilcoxon test.

Genes encoding for UPEC iron-acquisition were also studied, including the outer membrane ferric yersiniabactin/enterobactin importers (*fuyA/fepA*), the yersiniabactin (*ybtS*) and enterobactin synthesis (*entD*) and the heme transport (*chuA*) (Fig. 6A). Regardless of the condition, most of the genes were upregulated (except *fepA*) at the UC site for the planktonic and adhered UPEC. This is due to a large amount of iron present in the UC medium. Per the gene’s expression, their corresponding metabolites (e.g., yersiniabactin, enterobactin) were found in UC medium under both treatment and control conditions (Fig. 6B).

A significant overexpression of *fuyA* (*P ≤ *0.05), *chuA* and *entD* (*P ≤ *0.01) genes were found in gut dialysate under PAC treatment, while genes remained downregulated under the control condition (Fig. 6C). This was true in the presence of the microbial metabolome, while in its absence, they remained downregulated like the control condition (Fig. 6A). However, the metallophores were not detected in UPEC dialysate.

## DISCUSSION

UPEC display remarkable capacity to metabolically adapt to diverse nutritional/physicochemical environments as found in the gut and the bladder (18, 19). Considering the limited information available on UPEC spatial ecology-pathophysiology, this study explored, in a unique fashion, the effects of metabolized PAC-rich cranberry extract on growth and virulence genes expression of UTI89 in contrasting ecosystems (i.e., transverse colon and urothelium). These ecosystems were mimicked in a novel model combining a colonic fermentation system interacting with a dialysis cassette device containing UPEC and a 3D tissue-engineered urothelium.

### Tracking UPEC virulence in its different ecological niches.

To our knowledge, our study reveals for the first time that UPEC virulence genes activation is independent of the urothelium infection site “urovirulence” (20). Remarkably, most of the studied genes encoding adhesins and toxins were already overexpressed upstream in the colonic non-treated microbiota. At this site, UPEC is recognized as being inoffensive (2, 3). However, our work, supports the idea that virulence factors cannot directly predict UTI symptoms but presumably confer multiple other functions (21). Since the SHIME only mimics the microbial interactions and physicochemical parameters of the luminal part of the colon but does not include intestinal epithelial cells, we cannot truly claim UPEC as enterovirulent. Such concept of UPEC enterovirulence should be specifically tested in the presence of intestinal epithelial cells, as recently discussed in the work of Schultz et al., using an intestinal model of Caco-2 cells (22). The authors have shown that the strain UPEC 536, positive for the hemolysin toxin production (HlyA+), induced epithelial barrier dysfunction by compromising tight junctions as potentiator of the leaky gut syndrome (22). Our work concords with that of Schulz et al. (22), highlighting the importance of studying UPEC upstream the famous urovirulent stage.

### Cranberry PAC microbial-derived metabolites impede UPEC virulence genes activation: dealing with donor-dependent responses for a more personalized approach.

The European Association of Urology has not yet formally recommended the use of PAC-rich cranberry extracts against UTI due to inconsistencies among human clinical trials and knowledge gaps in the mechanisms of action of PAC in the urinary tract (15, 23). Our study attempted therefore to provide new insights on the presumed mode of actions, and specifically assess the molecular mechanisms at play in the gut reservoir in the presence of a whole and natural microbiota. We demonstrated that PAC-rich cranberry extract microbial metabolites significantly impede the activation of UPEC virulence genes at early stage in the gut reservoir (*hlyA*, *cnf1*, *papG*, *fimH*, *Sfa*). Such gene inhibitions under PAC treatment tended also to be seen in the urothelium habitat (planktonic and adhered UPEC), although the effect was not significant. The lack of statistical difference was due to controversial response between donors in UPEC genes expression. The genes *hlyA*, *cnf1*, *fimH*, and *eaeH* remained upregulated in donor A under both control and PAC treatment, while an opposite profile was observed in donor B. Hence, the interindividual variability in genes expression at the urothelium site might be attributed to upstream differences in intestinal microbiota composition (e.g., age, gender, race, diet, health status, environmental exposure, etc.) among individuals (6) and its ensuing PAC metabolism. Indeed, cranberry PAC-polyphenols underwent extensive microbial metabolism during colonic digestion, triggering a wide range of phenolic metabolites, as observed here and in previous studies (12–17). However, contrasting microbiota between individuals can lead to the selective release of specific bioavailable microbial-derived PAC metabolites (14, 24–27). In our study, among the spectrum of PAC metabolites produced under colonic fermentation, donor B was producing 5-(3′,4′-dihydroxyphenyl)-γ-valerolactone contrary to donor A. Such variability between donors, therefore, indicates the need to profile individuals’ gut metabolomes as metabotypes when conducting clinical interventions, to define responder(s) class(es) in a perspective of targeted personalized nutrition (28).

Interestingly, compared with donor A, donor B exhibited an overall better response to the PAC treatment, that is, a marked virulence genes downregulation and a reduced UPEC adhesion onto UC. The antiadhesive activity of phenyl-γ-valerolactone against UPEC was previously reported in bladder epithelial cells (17), but no studies have assessed its role on the anti-virulent activity of UPEC. Most studies only assessed the ability of PAC and its corresponding metabolites to inhibit bacterial adherence/invasion to UC, biofilm formation, or clinical symptoms reduction (6, 12–15). They briefly described the anti-virulent properties of PAC in simple static culture medium (29–31). For instance, one study reported that cranberry compounds could inhibit UPEC motility via downregulation of the *fliC* gene (30). In a more extensive microarray study, UPEC transcriptomic profiles were assessed in simple cultures supplemented with cranberry PAC alone or in combination with propolis (31). Both the PAC treatment and the combination downregulated expression of genes involved in UPEC adhesion, motility, and biofilm formation (31). Although these results are in accordance with our observations, our work is the only one assessing UPEC virulence in a natural luminal microbiota environment with its full metabolome complement, therefore reproducing more accurately the true human gut conditions. Finally, the observed modulation of virulence genes expression under PAC treatment does not necessarily indicates a modulation at protein level. Indeed, post-transcriptional and post-translational regulations are additional key steps that we must consider in future studies by using proteomic tools.

### Additional mode of action of cranberry PAC on UPEC: metallophore encoding genes interaction.

Metallophore encoding genes are additional key survival genes for UPEC, standing at midway between virulence and fitness pathways. UPEC encounter iron limitation within the host, such as in the urinary tract. Cranberry is also known as an iron chelator strongly limiting bacterial availability (30, 32). To ensure adequate intracellular iron levels, UPEC upregulates the expression of genes involved in iron acquisition (31, 33). Such iron-acquisition genes upregulation mechanism is confirmed in our study reproducing a complex colonic environment under PAC treatment compared to the control. Indeed, UPEC display a capacity to adapt to the presence of cranberry PAC by upregulating iron acquisition systems and reducing iron storage, as reported by Ranfaing et al. (31). Finally, in the urothelium, most of the iron-acquisition genes were upregulated under both conditions. The urothelium was however not the best model to study metallophore action since the medium was already supplemented with iron to maintain UC growth.

### Beyond PAC microbial-derived metabolites.

In addition to the PAC metabolites which appear to alter UPEC virulence genes, one should not overlook the role of SCFA as key fermentation metabolites affecting bacterial physiology. In our study, the 14-day treatment shaped the gut microbiota in a donor-dependent way. Yet, such changes resulted in a clear shift of SCFA production marked by a significant increase of propionate and butyrate for both donors. Such SCFA increase can be partly explained through the bloom of Akkermansia muciniphila in the two donors, a species recognized as propionate and butyrate producer (34, 35). Several studies have reported the role of SCFA in the modulation of enteric pathogens by lowering intraluminal pH and the capacity of SCFA to prime the virulence genes (36). However, there is so far no consensus regarding the SCFA role as virulence enhancer or inhibitor, which appear to be pathogen specific (37, 38). In our experimental conditions, the change of colonic pH cannot be observed since the pH was controlled. In addition, it is unlikely that the UPEC virulence genes downregulation under PAC treatment was associated to the increased production of propionate/butyrate. Indeed, in the control condition performed without microbiota, and thus without SCFA, no change in the expression profiles was seen compared to the treated microbiota.

### Conclusion.

Cranberry PAC microbial-derived metabolites could be of great public health significance and may represent a nonantibiotic alternative for UTI prophylaxis. In addition to reducing UPEC growth, we unraveled that microbial cranberry PAC metabolites display a remarkable capacity to blunt activation of genes encoding toxin production and adhesin/invasins in the gut reservoir and in coculture on the urothelium, in a donor-dependent manner. We should obviously consider interindividual variability in PAC microbial metabolism to identify responsive metabotypes in the patients and stratify them according to the specific PAC metabolizing microbiota (39). This approach would be of great interest to design UPEC anti-virulence prophylaxis in a personalized fashion, based on microbial or metabolomic markers of response. Further investigations integrating multi-stage microenvironments such as the coculturing of urothelium with bladder microvascular endothelial cells exposed to urine as well as microbiome and urobiome interactions would also provide more insights on the PAC metabolites underlying mechanisms of action.

## MATERIALS AND METHODS

### Strain.

UPEC strain used in this study was SLC-719 O18:K1:H7, an isolate derivative of the human cystitis strain UTI89, carrying the fluorescent protein vsfGFP-9 (40, 41) which possesses virulence genes encoding for the type 1 fimbrial adhesin (*fimH*), attaching and effacing protein (*eaeH*), S fimbriae (*sfa*), pili associated pyelonephritis (*papG*), curlin major subunit (*csgA*), as well as for the toxins alpha hemolysin (*hlyA*) and cytotoxic necrotizing factor 1 (*cnf1*). UPEC was routinely grown under agitation (37°C, 150 rpm, overnight) in Luria Bertani (LB) broth (BD Difco, USA). The number of cultivable UPEC was determined by direct plating onto LB agar and expressed in log_10_ CFU (CFU)/mL.

### Fermentation system.

The TWIN-SHIME (Prodigest, Belgium) is a simulator of the human intestinal microbial ecosystem with two anaerobic SHIME units operated in parallel, in semi-continuous mode (42). In this study, a SHIME unit consisted of a stomach/small intestine vessel to reproduce gastric digestion of a standardized nutritional medium pancreatic/bile juice delivery, followed by a proximal colon and a transverse colon vessel. Two healthy fecal donors, one in each SHIME unit, inoculated the proximal and transverse colons to capture inter-individual variability in microbiota composition and PAC metabolism. Consent for fecal donation was obtained under registration number 2019-312 (Laval University, Canada). Procedure for fecal inoculum preparation was previously described (43). After a 2-week stabilization period, a 7-day control diet followed by a 14-day treatment was applied to the system (Fig. 1A). Treatment consisted in the supplementation of a PAC-rich cranberry extract, containing 86.8 mg PAC/day/donor (Urophenol-Diana Food, France) in the SHIME stomach for 14 days. Extract characterization is provided in Table S1 in the supplemental material.

### Microbial community analysis.

Following the DNA extraction of the SHIME samples as previously described (44), next-generation *16S* rRNA gene amplicon sequencing of the V3-V4 region (341F-805R) was performed at IBIS (Institut de Biologie Intégrative et des Systèmes, Laval University, Canada), on an Illumina MiSeq platform with Illumina V3 chemistry using the 600-cycle reagent kit (Illumina, USA). The Divisive Amplicon Denoising Algorithm (DADA2) workflow implemented in the *dada2* R package (version 1.18.0) was employed to identify Amplicons Sequences Variants (ASVs) (45). The advantage of using *dada2* over traditional clustering methods is that it determines the exact sequences based on an error model for the sequencing run, resolving as little as one nucleotide difference. The reads were merged if they overlapped precisely, and an ASVs table was constructed, recording the number of times each ASV was observed in each sample. Default parameters were used to estimate error parameters using *learnErrors*, and chimeras were removed using *removeBimeraDenova* (method = "consensus”). ASVs sequences were assigned taxonomy using the most recent SILVA taxonomic database (SILVA SSURef 138.1 NR, March 2021) as a reference data set (46). A phyloseq data object was created using the *phyloseq* package in R (47). Unassigned taxa and singletons were removed. Rarefaction curves were constructed to ensure that the samples were sequenced at sufficient depth (48). Relative abundances of microbial genera were plotted using the *ggplot2* packageafter transforming abundance data into relative abundances.

### Short Chain Fatty Acids (SCFA) analysis.

Colonic samples from SHIME were centrifuged 8 min at 14000g at 4°C. Supernatants were stored at –20°C until analysis. Aliquots of 125 μL of sample were diluted with H_2_O Milli-Q water, spiked with a solution containing an internal standard (4-methylvaleric acid, Sigma, Canada) and H_3_PO_4_ (Fisher, Canada), 10% to obtain a pH of 2. A volume of methyl tert-butyl ether (Sigma, Canada), equivalent to the volume of diluted sample was added to extract SCFA by vortexing2min. Samples were then centrifuged 10min at 14000g at 4°C and organic phases were transferred to a glass vial. SCFA analysis was performed on a GC-FID system (Shimadzu, Japan), consisting of a GC2010 Plus gas chromatograph equipped with an AOC-20s auto-sampler, an AOC-20i auto-injector and a flame ionization detector. The system was controlled by GC solution software. One microliter of organic phase was injected in a split mode into a Nukol capillary GC column (30m ×0.25mm id, 0.25 μM film thickness, Supelco analytical) and hydrogen was used as carrier gas. The injector and detector were set at 250°C. The oven temperature was initially programmed at 60°C, then increased to 200°C at 12° C/min and hold at this temperature for 2 min. SCFA were quantified using a 5-points calibration curve prepared with a mix of standards (acetic acid, propionic acid, butyric acid, isobutyric acid, valeric acid and isovaleric acid, Sigma, Canada) extracted following the same procedure as samples.

### Dialysis cassette confined UPEC exposure to SHIME metabolome.

Slide-A-Lyzer Dialysis Cassettes 10 kDa pores (ThermoFisher Scientific, Canada) were used to test UPEC short-term exposure to the colonic metabolome of the TWIN-SHIME. This device maintains UPEC confined within the cassette and avoids the contamination of the whole reactor while allowing recovery of the pathogen for further analysis. The semipermeable membrane contains 10 kDa pores that are large enough to let colonic metabolic compounds pass through but restrict the passage of bacteria. UPEC pre-culture was resuspended in saline buffer and injected into the cassette at a dose of 7log_10_ CFU/3 mL (Fig. 1B). Cassettes containing UPEC were introduced in the transverse colon treated with or without PAC extract until the endpoint of diffusion of 2 h. The transverse colon was chosen for UPEC reservoir and PAC catabolism (49–51). An additional condition without microbiota was tested separately in anaerobic jars containing only the nutritional medium and pancreatic juice adjusted at the pH of the transverse colon. In each condition the cassettes were removed from the reactors after a 2-h incubation. Dialysates containing UPEC were collected with a syringe and aliquoted. To count the number of UPEC cells remaining after dialysis, 100 μL of dialysates were taken apart and serially diluted in saline water and plated onto LB agar (overnight incubation at 37°C). In parallel, the remaining 2.9 mL of dialysate samples were divided in two tubes and both pelleted 10 min at 14000g at 4°C. One tube of UPEC pellet was resuspended in 500 μL RNA*later* (Thermo Fisher Scientific, USA) and stored at –80°C prior to RNA extraction, while the second tube of UPEC pellet was stored and preserved at –80°C in glycerol 10% until cell culture.

### 3D tissue-engineered urothelium culture and infection.

Bladder mesenchymal primary cells (BMC) and urothelial cells (UC) were extracted from a bladder biopsy (52). BMC and UC were cultured in media as previously described (53) and incubated at 37°C in a humidified 8% CO_2_ atmosphere. A suspension of 1,500,000 BMCs in 3 mL BMC medium was mixed with 3 mL of 5 mg/mL PureCol-EZ collagen solution (Advanced Biomatrix, USA) and distributed in 6 wells (including a paper anchorage) to produce 1 cm^3^ of cellularized collagen gel. The day after, 200,000 UCs were seeded on top of the collagen gel. Constructs were cultivated for 7 days in UC medium supplemented with 50 μ/mL ascorbate (Sigma, Canada). Engineered tissues were then put at the air/liquid interface using a specific device (54) and cultivated in UC medium for another 21 days with ascorbate (Fig. S1 in the supplemental material). On the day of infection, an antibiotic-free UC medium was used. UC were infected with 1 mL UPEC dialysates containing 6log10 CFU/mL, in duplicate for each condition (Fig. 1C). After 6 h, the medium containing non-adhered (planktonic) bacteria was collected, and tissues were rinsed three times using 2 mL PBS. Tissues were fixed 10 min in pure acetone at –20°C before performing immunolabelling. 100 μL of UC medium (planktonic UPEC) and rinsed tissue (adhered UPEC) were serially diluted in saline water and plated onto LB agar (overnight incubation at 37°C). The number of cultivable UPEC was expressed in log_10_ CFU (CFU)/mL.

### Immunolabelling.

Punches of 4 mm from fixed tissues were rinsed in PBS and incubated 1 h with PBS/BSA 1% before sequential 24 h incubation with a primary antibody AE1/AE3 diluted 1/200 and a mix of secondary antibodies Alexa Fluor 594 (Abcam, Canada) diluted 1/400 with Hoechst 33258 diluted 1/200 (Sigma, Canada). Between each step, tissues were washed several times with PBS, and finally covered with mounting medium and coverslips. The slides were observed with a Zeiss LSM 800 laser-scanning confocal microscopy system (Zeiss, Canada).

### Isolation of UPEC RNA and qRT-PCR.

RNA was isolated from UPEC dialysates, adherent and planktonic UPEC in UC, using TRIzol method (Invitrogen, USA) (43) and MICROBEnrich kit (Ambion, USA). UPEC mRNA was converted to cDNA using PrimeScript RT Kit (TaKaRa, Japan). Expression of UPEC genes was quantified on StepOnePlus real-time PCR system (Applied Biosystems, USA) and normalized to the housekeeping genes *arcA* and *gapA*. Primers used are described in Table S2 in the supplemental material.

### Semi-targeted metabolomics for relative quantification of PAC secondary metabolites and metallophores.

Samples preparation consisted in the addition of 50 μL of cold methanol (LC-MS grade) to 50 μL colonic or UC samples to stop the metabolic activity. Ten microliters of internal standards solution (in 1:1 methanol/water) were added at a final concentration of 10 ppm for gallic acid-d_2_, succinic acid-d_6_, trans-cinnamic acid-d_5_, L-tryptophan-d_5_, glycocholic acid-d_4_, l-leucine-d_7_, 4-hydroxybenzoic acid-d_4_, methyl 4-hydroxybenzoate-d_4_; except for caffeine methyl-d_3_ (1 ppm) and n-dodecylphosphocholine-d_38_ (0,1 ppm). Samples were then filtered using micro-spin filter tubes (Nylon 0.22 μm, Canadian Life Science, Canada) by centrifuging them for 10 min at 10,000 g before LC-MS analysis. Relative quantification of PAC secondary metabolites and metallophores was performed on UPLC-Q-ToF (Acquity I-Class coupled with a Synapt G2-Si, Waters, USA).

The chromatographic method was adapted from Mena et al., 2017 (17). Briefly, 5 μL of samples were injected onto an ACQUITY UPLC HSS T3 column (2.1 × 100 mm, 1.8 μm) (Waters, USA) with an ACQUITY UPLC HSS T3 VanGuard pre-column (2.1 × 5 mm, 1.8 μm) (Waters, USA) heated to 30°C. The mobile phases were water (A) and acetonitrile (B), both acidified with 0.1% formic acid, and the flow rate was 0.3 mL/min. The gradient was as follows: 0–0.5 min: 2% B, 0.5–9 min: 2–45% B, 9–9.5 min: 45–80% B, 9.5–15.5 min: 80% B, 15.5–16 min: 80–2% B and 16–22 min: 2% B. Samples were kept at 4°C in the autosampler compartment. For the MS analysis, data were acquired by MS^E^ in both positive and negative electrospray ionization and resolution mode (resolution ≈ 25 000) with a scan time of 0.2 s. Each function was collected with a scan range (*m/z*) of 50 to 1200. A collision energy ramp of 20 to 50 V was applied in the high energy function. The source parameters were as follows: capillary voltage, +1,20 kV in positive ionization and -2,40 kV in negative ionization; source temperature, 120°C; desolvation temperature, 400°C; cone gas flow, 50 L/h and desolvation gas, 800 L/h. Leucine-enkephaline (200 pg/μL) was infused at a flow rate of 10 μL/min for use as internal mass standard. Each sample was injected in triplicate.

Metabolite identification was confirmed using an UHPLC-Orbitrap (Vanquish Flex coupled with Orbitrap Tribrid Fusion, Thermo Scientific, USA) (Tables S3 and S4 in the supplemental material). Chromatographic conditions were the same as for analysis with UPLC-QToF. Data-dependant MS^2^ with an inclusion list was performed to confirm the identification of metabolites. The method started with a survey scan in the Orbitrap analyzer in positive ionization mode with a resolution of 120 000. The scan range (*m/z*) was 100 to 1,000, and the RF lens was set to 60%. After, ions were isolated by the quadrupole with a window size of 1.6 *m/z* and fragmented in the HCD cell with stepped collision energy (20, 40 and 65%). Fragments were analyzed in the Orbitrap with a resolution of 30 000. For each MS survey scan, three MS^2^ scans were acquired. The source parameters were as follows: spray voltage, +3,500 V; sheath gas flow, 50 Arb; aux gas flow, 10 Arb; sweep gas flow, 1 Arb; ion transfer tube temperature, 325°C and vaporizer temperature, 350°C. All acquired data were corrected using the internal mass calibrant (EASY-IC^TM^). Data acquired with the UHPLC-Orbitrap were processed using Compound Discoverer. Progenesis QI and Skyline were used to process data from the UPLC-Q-ToF.

### Statistical analyses.

Statistical analyses were performed in R studio, version 4.0.4 (55), using the PMCMR package version 4.3. All formal hypothesis using non-parametric tests were conducted on the 5% significance level (*P ≤ *0.05). Friedman test with *post hoc* Wilcoxon test was used to compare control versus PAC treatment conditions.
