# Supplementary material for: UPEC Colonic-Virulence and Urovirulence Are Blunted by Proanthocyanidins-Rich Cranberry Extract Microbial Metabolites in a Gut Model and a 3D Tissue-Engineered Urothelium
Source: Microbiol Spectr. 2022 Aug 16;10(5):e02432-21. doi: 10.1128/spectrum.02432-21 (PMC9603664; doi:10.1128/spectrum.02432-21)
Supplement: Supplemental file 1 — Supplemental material. Download spectrum.02432-21-s0001.pdf, PDF file, 0.5 MB [file spectrum.02432-21-s0001.pdf]

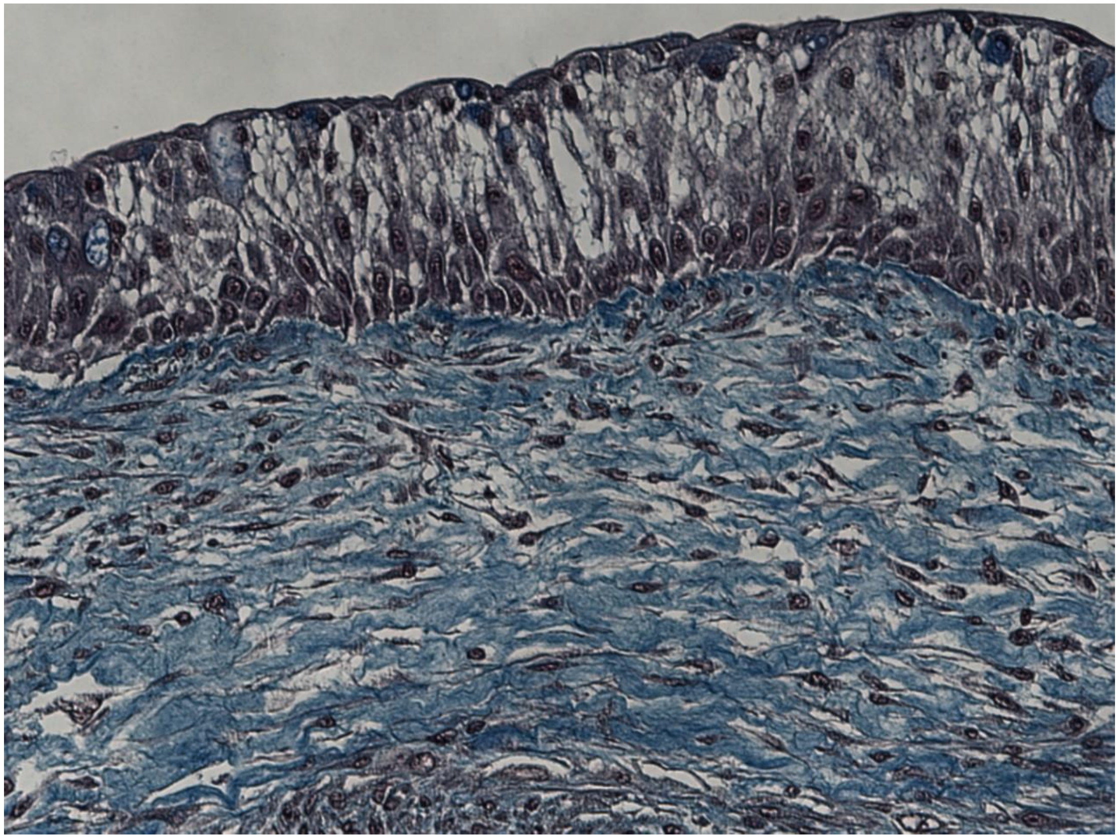

Supplementary Figure 1. Histology of a 3D human tissue-engineered bladder mucosa. Prior to UPEC infections, some tissues were fixed in formol 3.7% for 24h before to be included into paraffin. Tissues were sliced and stained using the Masson's trichrome protocol. The BMCs into the collagen gel formed the lamina propria whereas the BUCs seeded on the stromal part formed the urothelium. The three layers of the epithelium can be distinguished: basal lamina, intermediate cells (racket morphology) and umbrella cells (flat cells on the top).

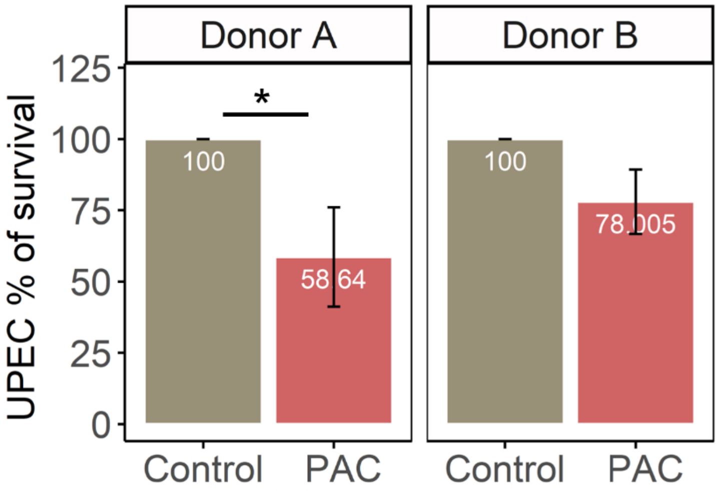

Supplementary Figure 2. Percentage of viable-culturable UPEC remaining in the dialysis cassettes after a 2-hour exposure with the PAC treated/non-treated metabolome of the transverse colon. Significant differences between PAC treatment and control are indicated with $p \leq 0.05$ (*), as determined by the Friedman post-hoc Wilcoxon test.

Supplementary Table 1. Characterization of PAC-rich cranberry extract by UPLC-UV-QToF and HPLC-UV

	Analyte	Concentration (mg/g) (mean ± standard deviation)		
Phenolic acid	Coumaric acid	1,285	±	0,018
	Coumaroyl-dihydromonotropein	0,754	±	0,009
	Chlorogenic acid	1,92	±	0,03
	Coumaroyl-hexoside - 1	0,333	±	0,005
	Coumaroyl-hexoside - 2	0,182	±	0,003
	Caffeoyl-hexoside	0,172	±	0,002
	Sinapoyl-hexoside	0,352	±	0,006
	Coumaroyl-monotropein	0,372	±	0,006
	Vanilloyl-hexoside	0,0533	±	0,0009
	Feruloyl-hexoside	0,209	±	0,003
	3,4-dihydroxybenzoic acid	ND	±	ND
	Hydroxybenzoyl-hexosyl-hexoside	0,1247	±	0,0019
	Dihydroxybenzoic acid - 2	0,116	±	0,004
	Dihydroxybenzoic acid - 3	0,0501	±	0,0006
	Dihydroxybenzoyl-hexoside	ND	±	ND
	Phenolic acids and derivatives total	5,93	±	0,09
Flavonols	Quercetin	2,9	±	0,04
	Myricetin	1,52	±	0,02
	Quercetin-3-galactoside	0,348	±	0,005
	Quercetin-3-rhamnoside	0,783	±	0,011
	Myricetin-hexoside	0,145	±	0,002
	Quercetin-pentoside - 1	0,54	±	0,03
	Quercetin-pentoside - 2	0,352	±	0,007
	Quercetin-pentoside - 3	0,827	±	0,013
	Quercetin-hydroxybenzoyl-hexoside	0,279	±	0,004
	Isorhamnetin	0,223	±	0,003
	Myricetin-pentoside 1	0,0865	±	0,0017
	Syringetin-hexoside	0,193	±	0,003
	Myricetin methyl	0,124	±	0,002
	Myricetin-pentoside - 2	0,1307	±	0,0014
	Myricetin-pentoside - 3	0,321	±	0,007
	Quercetin-deoxyhexoside	0,1034	±	0,0017
	Flavonols total	8,87	±	0,15

Anthocyanes	Cyanidin-3-galactoside	0,034	±	0,003
	Cyanidin-3-glucoside	0,069	±	0,006
	Cyanidin-3-arabinoside	0,75	±	0,03
	Peonidin-3-galactoside	0,063	±	0,006
	Peonidin-3-glucoside	0,156	±	0,006
	Peonidin-3-arabinoside	0,577	±	0,017
	Anthocyanes totaux	1,65	±	0,07
PAC	Catechin	0,182	±	0,004
	Epicatechin	1,31	±	0,02
	Flavan-3-ols total	1,49	±	0,02
	Procyanidin A2	3,19	±	0,06
	Procyanidin B2	ND	±	ND
	Trimer PAC A-Type (1x A) - 1	0,96	±	0,02
	Trimer PAC A-Type (1x A) - 2	1,55	±	0,03
	Trimer PAC B-Type - 1	6,65	±	0,1
	Trimer PAC B-Type - 2	1,32	±	0,02
	PAC total *	70,9	±	1,6
	Flavan-3-ols + PAC total	72,4	±	1,6
	Mean DP *	6	±	0,013

** Determined by phloroglucinolysis.*

Supplementary Table 2. UPEC primers specific to the UTI-89 strain SLC719

	Genes	Genbank accession number	Primers	Tm	Amplicon size	Reference
Adhesins-Invasins	***fimH***	CP000243.1	F-GTGCCAATTCCTCTTACCGTT	60°C	164 pb	Hojati et al. 2015
		Locus 903 pb	R-TGGAATAATCGTACCGTTGCG			
	eaeH	CP000243.1	F-AGTAACCGTGGTGGCTGATG	60°C	131 pb	This study
		Locus 4251 pb	R-CACTGATAGCGTTGCCATGC			
	sfa	CP000243.1	F-GCTGCAAATACGCACTGACC	60°C	199 pb	This study
		Locus 264 pb	R-AAGGTGTTGGTTCCCGGTAC			
	papG	CP000243.1	F-TTCACCATAGAGGCGACTGC	59°C	130 pb	This study
		Locus 961 pb	R-TTCGGTTGGTCTGGGTCATG			
	csgA	CP000243.1	F- TCTGGCAGGTGTTGTTCCTC	60°C	119 pb	This study
		Locus 421 pb	R-CAAGAGCAGAGTTACCGCCA			
Toxins	***hlyA***	CP000243.	F-GCAAATAAATTGCACTCAGCA	58°	142 pb	Madelung et al.

		1	G	C		2017
		Locus 3061 pb	R-CAAGGTCATTAAGGCTTGAACC			
	cnf1	CP000243.1	F-GGTATTCCGACGGGAGCATT	60°C	149 pb	This study
		Locus 3001 pb	R-ACTGACACTCACTCAAGCCG			

Supplementary Table 3. UPEC metallophores identification - positive ionisation

**Compound**	**RT Synapt (min)**	**$[M+H]^+$, *m/z* detected (Synapt)**	**RT Fusion (min)**	**$[M+H]^+$, *m/z* detected (Fusion)**	**Mass accuracy (mDa)**	**MS^2 fragments, *m/z* (Relative intensity)**	**Identification level**
**Yersianiabactin - Isomer 1**	10,24	482,1243	11,59	482,1237	-0,5	295.0573 (100) ; 190.0323 (42) ; 114.0370 (8)	2
**Yersianiabactin - Isomer 2**	10,48	482,1242	11,89	482,1239	-0,3	295.0570 (100) ; 190.0322 (49)	2
**Yersianiabactin-Fe(III)**	8,4	535,0359	10,08	535,0355	-0,1	302.9889 (100) ; 201.9620 (96) ; 188.0740 (77) ; 348.9767 (43)	2
**Enterobactin**	8,85	670,1482	10,56	670,1516	-0,4	224.0553 (100) ; 137.0231 (33) ; 447.1042 (23)	2

*Mass accuracy and MS^2 fragments are from Fusion data.

Supplementary Table 4. PAC metabolites and flavonol identification - negative ionisation

Class	Compound	RT Synapt (min)	[M-H]-, *m/z detected* (Synapt)	Mass accuracy (mDa)	MS^2 fragments, *m/z* (Relative intensity)	Identification level
**PAC metabolites**	Benzoic acid	7,19	121,0292	0,2	ID confirmed by analytical standard	1
	2-hydroxybenzoic acid	7,57	137,0242	0,3	ID confirmed by analytical standard	1
	3,4-dihydroxybenzoic acid	3,89	153,0192	0,4	ID confirmed by analytical standard	1
	3,5-dihydroxybenzoic acid	3,81	153,0192	0,4	ID confirmed by analytical standard	1
	2-(4′-hydroxyphenyl)acetic acid	5,04	151,0397	0,2	ID confirmed by analytical standard	1
	3-(3′-hydroxyphenyl)propanoic acid	6,28	165,0553	0,2	ID confirmed by analytical standard	1
	3-(4′-hydroxyphenyl)propanoic acid	5,87	165,0553	0,2	ID confirmed by analytical standard	1
	3-(3′,4′-dihydroxyphenyl)propanoic acid	4,91	181,0501	0	ID confirmed by analytical standard	1
	5-(3′-hydroxyphenyl)valeric acid	8,23	193,0868	-0,2	ID confirmed by analytical standard	1
	5-(3′,4′-dihydroxyphenyl)valeric acid	6,89	209,0814	-0,1	ID confirmed by analytical standard	1
	5-(3′,4′-dihydroxyphenyl)-γ-valerolactone	5,83	207,0653	-0,4	ID confirmed by analytical standard	1
	1-(Hydroxyphenyl)-3-(2″,4″,6″-trihydroxyphenyl)-2-propanol	6,36	275,0917	-0,2	123.0448 (100) ; 135.0449 (43) ; 151.0398 (25)	2
	1-(Dihydroxyphenyl)-3-(2″,4″,6″-trihydroxyphenyl)-2-propanol	5,33	291,0868	-0,1	137.0242 (100)	2
	Catechin	5,24	289,0710	-0,2	ID confirmed by analytical standard	1
	Epicatechin	4,77	289,0704	-0,8	ID confirmed by analytical standard	1
	Procyanidin A1	5,91	575,1194	0,4	ID confirmed by analytical standard	1
	Procyanidin A2	6,41	575,1207	1,7	ID confirmed by analytical standard	1
	Procyanidin B1	4,27	577,1367	2,1	ID confirmed by analytical standard	1
	Procyanidin B2	4,92	577,1366	2	ID confirmed by analytical standard	1

**Flavonol**	Quercetin	8,43	301,034	-0,8	ID confirmed by analytical standard	1

*Mass accuracy and MS2 fragments are from Synapt data.
